# Associations of home and neighborhood environments with children’s physical activity in the U.S.-based Neighborhood Impact on Kids (NIK) longitudinal cohort study

**DOI:** 10.1186/s12966-023-01415-3

**Published:** 2023-02-02

**Authors:** Alison Carver, Ester Cerin, Muhammad Akram, James F. Sallis, Kelli L. Cain, Lawrence D. Frank, Carrie M. Geremia, Terry L. Conway, Karen Glanz, Brian E. Saelens

**Affiliations:** 1 Peninsula Clinical School, Monash University, Frankston, VIC Australia; 2 Mary MacKillop Institute for Health Research, Australian Catholic University, Melbourne, VIC Australia; 3 School of Public Health, The University of Hong Kong, Hong Kong, China; 4 Herbert Wertheim School of Public Health and Human Longevity Science, University of California, San Diego, La Jolla, CA USA; 5 Dept. of Urban Studies and Planning, University of California, San Diego, La Jolla, CA USA; 6 Perelman School of Medicine and the School of Nursing, University of Pennsylvania, Philadelphia, PA USA; 7 Dept. of Pediatrics, University of Washington and Seattle Children’s Research Institute, Seattle, WA USA

**Keywords:** Recreation, Built environment, Moderator analysis, Context, Accelerometer

## Abstract

**Introduction:**

Physical activity is important for children’s health and well-being. Supportiveness for physical activity of home and neighborhood environments may affect children’s PA, but most studies are cross-sectional. We examined environmental predictors of change in children’s physical activity over two years.

**Methods:**

Data were from the longitudinal, observational cohort study, ‘Neighborhood Impact on Kids’. Participants were children (initially aged 6–12 years) and their parent/caregiver (*n* = 727 dyads) living in neighborhoods throughout San Diego County, California and King County (Seattle area), Washington, USA. Children’s moderate-to-vigorous physical activity (MVPA) was measured using accelerometers at T1 (Time 1 or baseline, 2007–2009) and T2, the two-year follow-up. At T1, parents survey-reported on physical activity (PA) equipment at home and demographics. Neighborhood environment was measured using spatial data in Geographic Information Systems (intersection density; park availability) and in-person audits (informal play space near home; park-based PA facilities; land use; support for walking/cycling). Generalized additive mixed models estimated total effects, then direct effects, of environmental attributes on MVPA at T1. Two-way moderating effects of child’s sex and age were examined at T1. To examine associations of environmental exposures with changes in MVPA, we estimated interaction effects of environmental attributes on the association between time and MVPA.

**Results:**

On average, children accumulated 146 min/day (standard deviation or SD = 53) of MVPA at T1, and 113 (SD = 58) min/day at T2. There were no significant total or direct effects of environmental attributes on MVPA at T1, and no significant two-way interaction effects of child’s age and sex for T1 MVPA. Having informal play spaces proximal to home with more amenities was associated with less MVPA decline from T1 to T2. Higher residential density, higher land use mix, and higher number of PA facilities in nearby parks were unexpectedly associated with greater MVPA decline.

**Conclusion:**

Higher quality informal play spaces close to home may help offset declines in MVPA during middle childhood, as they may promote unstructured active play with opportunities for parental or neighbor surveillance. Unexpectedly, environmental factors consistent with higher walkability were associated with greater declines in children’s MVPA. As physical activity differs across the lifespan, so may environmental factors that facilitate it.

**Supplementary Information:**

The online version contains supplementary material available at 10.1186/s12966-023-01415-3.

## Background

Across the lifespan regular physical activity (PA) provides many well-documented health benefits that include reducing risk factors for cardiometabolic diseases and some cancers [1]. Childhood is a key developmental life stage during which regular PA is instrumental in the promotion of optimal health, including bone mineral density and healthy weight status [1, 2]. PA levels have been shown to track from childhood into adulthood, so promotion of PA during childhood could benefit long-term health [3–5]. Globally, however, fewer than 25% of school-aged children engage in recommended levels of PA (i.e., at least one hour of moderate-to-vigorous-intensity physical activity or MVPA per day) [1, 6, 7], and age-related declines in PA are evident through late childhood and adolescence [4, 7].

Ecological models of health behaviors [8] lead to predictions that children’s physical activity is influenced by multiple nested layers of variables: at the individual level (e.g. child’s age, sex), social level (e.g. parental modelling of PA), neighborhood level (e.g. access to playgrounds), as well as at the policy level (e.g. mandatory inclusion of bicycle paths alongside new road infrastructure) [9, 10]. Several systematic reviews have explored factors associated with children’s PA within multiple contexts, i.e. individual, home/family and neighborhood [9, 11, 12]. In particular, the potential of the home environment as a setting for PA has attracted some research interest [13, 14]. For example, a systematic review [13] identified limited evidence of associations of PA equipment in the home with children’s PA. However, few longitudinal studies were included in that review, and few studies incorporated device-measured PA [13]. Another systematic review [14] found that having static PA equipment such as treadmills and exercise bikes in the home was associated with adolescents girls’ PA, while mobile PA equipment (e.g. rackets, bats, balls) was associated with younger children’s PA, with evidence of moderation by sex of child [14]. Once again, few studies with prospective/longitudinal designs were available to be included in that review [14]. A more recent study [15] of children aged approximately 11 years in 6^th^ grade of school focused on the after-school period from 3.30 – 6.00 pm, which has been identified as a ‘critical window’ for children’s PA [16]. Availability of PA equipment at home was associated with after-school MVPA in boys but not in girls [15]. An even more recent longitudinal study [17] that recorded self-reported PA of children in grade 5 and followed them up in grades 6,7, and 9 found positive associations between having home-based PA equipment and home-based PA. However, the strength of these associations diminished over time [17].

Beyond the home and school environments where children spend much of their waking hours, the neighborhood is a pivotal setting for their PA due to its proximity to home and accessibility by active transport, such as walking or cycling – two habitual sources of PA for children [18]. Parks and playgrounds tend to be accessible free of charge and are, therefore, particularly important for promoting PA through active play in socio-economically disadvantaged areas [10, 19]. However, the quality of facilities in parks and playgrounds may vary with area-level socioeconomic status (SES) [20]. In addition to providing settings for informal and unstructured physical activity, some neighborhoods may include dedicated sports facilities that offer opportunities for structured sports classes and team sports [21].

Some neighborhoods are more supportive of PA than others, and those that are conducive to walking to destinations, in particular, are considered to be ‘walkable’ [22]. Over the last decade research methods have been developed to quantify ‘walkability’, which is typically a composite measure that includes built environmental variables of residential density, land use mix and street connectivity [22–24]. Increasingly, the importance of walkable neighborhoods is recognized for the promotion of physical activity (through walking) and health-related benefits [24], as well as for reducing carbon emissions if short trips are made on foot rather than by car [23]. When investigating the potential influence of the neighborhood on children’s PA, child-specific destinations such as schools and playgrounds should be examined in conjunction with neighborhood walkability [25].

There is a need for broader research that investigates diverse aspects of the built environment that may impact overall PA. This research should include household structure and the interactions between neighborhood environments with parental and youth travel and activity patterns. Since the turn of the millennium, there has been much focus on environmental predictors of children’s active transport to school and other destinations [18, 26], as well as their independent mobility (i.e. freedom to walk/cycle from place to place without adult accompaniment) [27, 28] and active free play [29]. There have been audits of streetscapes near schools [30], playgrounds and recreational facilities [31], as well as examination of associations between road safety infrastructure and children’s PA [32]. Studies of children’s PA have investigated crime and personal safety as these may impact the extent to which parents allow their children to play in their neighborhood [33, 34]. Though a multiplicity of environmental settings and attributes are proposed to work together to influence physical activity [35], there is a paucity of studies that examine a broad range of home and neighborhood environmental characteristics that support diverse aspects of children’s PA such as active transport, free play and sports, with a particular deficit in studies of associations between environmental characteristics and change in children’s PA.

The aims of the present study were to examine:

1. cross-sectional associations of a wide range of household and neighborhood environmental characteristics with children’s PA;
2. associations of baseline household and neighborhood environmental characteristics with change in PA over two years among children aged 6–12 years at baseline.

## Methods

Data were drawn from the ‘Neighborhood Impact on Kids’ [NIK] longitudinal study which had a primary focus of identifying potential obesogenic environments and their impact on children’s weight status and behaviors related to nutrition and PA [36]. Details are published previously of selection of neighborhoods, operationalized here as census block.

groups in King County, Seattle area, Washington, and San Diego County, California [37, 38]. Census block groups within these counties were stratified first as “high” (favorable) or “low” (unfavorable) according to their supportiveness of PA, and then in a similar way for their supportiveness of healthy nutrition behaviors [37]. Supportiveness for PA was categorized according to walkability and access to high-quality parks, while supportiveness for nutrition was categorized according to availability of supermarkets for healthy food and fast food outlets for less healthy food [37]. This resulted in four categories of neighborhoods: low on both measures; low PA and high nutrition; high PA and low nutrition; and high on both measures [37]. Note that nutrition is not a focus of the current study.

A marketing company was commissioned to provide names, addresses and contact details of families with children aged 6–12 years residing in the selected census block groups. Households were randomly selected and contacted. Among 944 families who were eligible to participate, 727 did so. More specifically, children aged 6 to 12 years and one parent/caretaker living in selected block groups (727 child-parent dyads) were recruited between September 2007 and January 2009 (baseline or T1) for this cohort study [37]. Among these child-parent dyads, 646 remained eligible and participated at the two-year follow-up (T2).

Eligibility conditions for participation included residence in one of the selected census block groups (with the child-parent dyad spending five or more days per week there) and the child being physically able to engage in MVPA based on parent report. Children were excluded if they had a medical condition related to obesity or their growth, or had a psychiatric or eating disorder [37]. The same children and parent/caregiver were contacted two years later to complete the follow-up assessment (T2; September 2009 – February 2011), with the average length of follow-up being 23.7 months (standard deviation 1.9). More details about participant recruitment, enrollment, and differences between the T1 and T2 sample characteristics are provided elsewhere [37]. The sample size for NIK was selected to detect meaningful differences in weight status (and change therein) between children living in neighborhoods with different PA and nutrition environment profiles. The present analyses should be considered exploratory.

The NIK study was approved by Institutional Review Boards at Seattle Children’s Hospital, San Diego State University, and Emory University. Parents provided written consent and children assented to participate. Ethics clearance for the present secondary analysis of non-identifiable NIK data was granted by Australian Catholic University Human Research Ethics Committee (reference 2020-199N).

### Measures

#### Socio-demographics

At baseline (T1), parents were asked to complete a survey (either on paper or online), reporting their age, sex, highest level of education, marital status, number of children in the household, motor vehicles and driver’s licenses per household, city of residence, length of residence at current address; as well as the sex, age and ethnicity of their child participating in the study. To assess area-level SES, the median household income for each census block was extracted from US Census 2000 data.

#### Outcome measure – physical activity

Children’s PA was device-measured at T1 and two-year follow-up (T2) using the GT1M ActiGraph accelerometer (ActiGraph LLC, Pensacola, Florida) with 30-s epochs. Data collected by the uniaxial GT1M accelerometer has been shown to be comparable with that collected by the more recent triaxial GT3X accelerometer using the uniaxial mode, in standardized and free-living conditions [39]. Children were asked to wear the accelerometer for seven consecutive days, and re-wear was requested if fewer than six days’ worth of data including at least one weekend day were recorded with a minimum of 10 waking hours per day. For accelerometer scoring, a valid day was considered having 8 or more valid hours. Valid hours were defined as having no periods of 20 or more consecutive minutes of zero activity counts (designated as non-wear hours), with this criteria recommended by others [40]. Time spent in moderate-to-vigorous physical activity (MVPA) at each time-point was determined using age-based cut-points at three metabolic equivalents (METs) developed by Freedson and colleagues and validated for estimating children’s MVPA [41]. Activity was scored across all hours (not only valid hours) of valid days. If children had fewer than three valid days’ worth of accelerometer data at either time-point, their data were excluded from analyses.

#### Exposure measures

Objective spatial measures of the built environment were gathered using Geographic Information System (GIS) software, (Arc-GIS 9.3; Environmental Systems Research Institute, Inc., Redlands, CA, 2008). These included three variables considered to be components of walkability: intersection density, land use mix and residential density. Intersection density (i.e. number of intersections per km^2^) was computed using GIS and road network data for each census block group and a surrounding ‘shadow buffer’ of 0.25 miles (approximately 400 m) distance, to account for households on the edges of census block group whose environments may have been affected by adjoining census block group [38]. Land-use mix was operationalized here as the score for the ‘Positive destinations and land use’ subscale from the Microscale Audit of Pedestrian Streetscapes (MAPS), a physical, observational audit by trained research staff of the pedestrian environment within 0.25 mile (approximately 400 m) of the child’s home, in the direction of their school or other selected destinations [42]. Among these destinations were schools, shops, services, parks and recreation facilities [42]. Residential density was operationalized here as another MAPS subscale used to indicate the main type of housing building/residence in the audited area with possible values: 0 ‘commercial’, 1 ‘single family homes’, 2 ‘multi-family homes’, 3’apartments over retail only’ [42]. A further MAPS subscale was used to measure positive characteristics of the neighborhood for promoting active transport (AT). This positive AT score has possible values ranging from 0–44 (with higher scores indicating more support for AT) and was the sum of sub-scores for positive crossings, positive segments and positive streetscape [43].

Data on public parks in King County and San Diego County were gathered using a broad range of printed data, such as government lists of parks, locations and facilities, as well as digital sources, such as GIS spatial data including park perimeters and land parcels, and aerial photography [38]. After incorporating all these park data into GIS, the number of parks within 1 km of each child’s residence was calculated, regardless of whether the park was inside or outside of the child’s census block group [38].

Audits of park quality were conducted by research staff using the Environmental Assessment of Public Recreational Spaces (EAPRS) instrument [44] while visiting parks within the child’s census block group. This audit tool is used to assess the quality, cleanliness and condition of park features that encourage physical activity; for example trails, basketball courts and playground equipment, as well as supporting infrastructure such as toilets and bicycle racks [44]. In the present analysis, only the count of physical activity facilities available within a park was used, along with the number of parks within (or overlapping with) a child’s block group. For each child a ‘park PA facilities score’ variable was created by summing the PA facilities scores for all parks within or adjacent to the child’s census block group. If there were no parks present within or adjacent to the child’s census block group then a value of zero was assigned to the child’s park PA facilities score.

Research staff conducted in-person audits of local “informal” (i.e., not designated parks) play spaces within each child’s neighborhood using the Informal Play Space audit tool developed for the NIK Study (Supplementary file 1). These spaces had to be < 300 feet (91 m) from the child’s residence (boundary), adequately sized (> 500 square feet (46 m^2^) to allow for active play), and open and available for play (e.g., not structured or signed to prevent children playing). In addition to assessing the presence of play equipment and overall quality of the play space, this tool was used to record the visibility of the play space from the child’s residence and from other residences. The ability for parents to view a play space from home is important to promote spontaneous independent play among older children [45]. Further, the visibility of the play space from other residences can promote perceived and actual safety through discrete surveillance known as ‘eyes on the street’ [46]. The number of informal play spaces within each child’s census block group was computed as well as a quality score for each play space. Scores for all informal play spaces were summed to give a total play space score for the child’s census block group with possible values ranging from 0–47 (with higher scores indicating more support for active play).

Using the survey described above, parents reported their reasons for choosing to live in their current neighborhood, which was intended as a measure of neighborhood self-selection. In particular, parents were asked to rate the importance (on a five-point Likert scale ranging from 1 ‘Not at all important’ to 5 ‘Very important’) of the following 16 neighborhood characteristics: [1] affordability; [2] other children living nearby; proximity to [3] parks, [4] workplace/school, [5] public transport, [6] shops and services, [7] recreational facilities, [8] grocery stores and restaurants, [9] healthcare facilities and [10] entertainment/cultural facilities; [11] access to freeways/motorways; [12] ease of walking; [13] sense of community; [14] safety from crime; [15] quality of local schools and [16] other reason. Responses to these items were entered into principal components analysis with orthogonal varimax rotation, and three factors related to neighborhood self-selection were identified. These factors were neighborhood self-selection related to ‘leisure-time PA’, ‘transport-related PA’ and ‘safety and socioeconomic status’, with slight modification prior to their inclusion in analyses based on conceptual grounds (‘closeness to job or school’ was re-grouped with other transport-related PA items and ‘sense of community’ was re-grouped with ‘safety and socioeconomic status’ items). Cronbach’s α values for internal consistency of these groupings ranged from 0.65 (acceptable) to 0.83 (good).

Parents also reported on whether (‘1’) or not (‘0’) the following items of PA equipment were available for their child at home: bicycle; basketball hoop; skipping rope; active video games; sports equipment (e.g., balls, racquets, bats or sticks); swimming pool; skates/skateboard or scooter; and fixed equipment (e.g., swings). Test–retest for these items was reported previously (intraclass correlation coefficients: 0.53 – 0.83) [47]. Responses were summed to give a total for PA equipment at home with possible values ranging from 0–8.

Using survey items from the Neighborhood Environment Walkability Scale for Youth (NEWS-Y) [48], parents reported on their perceptions of safety regarding traffic (three items; e.g., traffic makes walking unpleasant) and crime in their neighborhood (five items; e.g., crime rates are high). Response options for each item ranged from 1 to 4 with higher numbers denoting more safety and were averaged for the corresponding traffic and crime scores. Cronbach’s α values for traffic and crime scores were previously demonstrated to be 0.60 and 0.82 for the two scales, respectively [49] indicating acceptable to good internal consistency.

### Data analytic plan

Descriptive statistics (means, standard deviations, percentages, and percentages of missing values) were computed, as appropriate, overall and by study site for all relevant variables. There were 20.9% of cases with missing data on at least one of the examined variables. The presence of missing data on specific variables was related to other variables included in the study, i.e., data were at least missing at random (MAR) rather than missing completely at random (MCAR) [50]). Consequently, ten imputed datasets were created for the main regression analyses as recommended by Rubin [50] and van Buuren [51]. Analyses based on complete data only when missing data are MAR can yield biased results, while analyses based on properly-conducted multiple imputations do not [50]. Multiple imputations were performed using chained equations (MICE) [51] accounting for clustering at census block group level and within-individual level arising from having two time-point assessments of PA (see under ‘*Outcome measure – physical activity’* in ‘Measures’ section above). The ten imputed datasets were created in R version 4.0.2 [52] using the package ‘mice’ version 3.11.0 and following the model-building and diagnostic procedures described by van Buuren [51].

To estimate how household and neighborhood environmental characteristics were associated with children’s MVPA, and to identify potential moderators of these associations, generalized additive mixed models (GAMMs; [53]) with Gaussian distribution were used. GAMMs are appropriate for modeling spatially-correlated and longitudinal data. They can also capture non-linear associations of unknown form. GAMMs were adjusted for potential confounders that were identified using a directed acyclic graph (DAG) which depicted the hypothesized relations between environmental characteristics, neighborhood self-selection factors, demographics, other covariates and children’s MVPA (Figure S2-1, Supplementary file 2). Using the DAG was beneficial for identifying covariates to be included in the statistical analyses to sufficiently control for potential confounders. We estimated first the total effects and then the direct effects of environmental attributes on child’s MVPA at T1. This is important because total effects quantify the overall potential effects of an environmental attribute on PA, while direct effects indicate the potential impact of an environmental variable on PA through mechanisms other than those included in the regression models. Subsequently, we examined the two-way moderating effects of child’s sex and age on the association between environmental attributes and child’s MVPA, adjusting for the same covariates as used in the direct-effect models. We did so because there are well-documented differences in children’s PA by age and sex [4, 6, 7]. All models also adjusted for clustering at census block group level and average accelerometer wear-time per valid day.

To examine the associations of baseline environmental variables with changes in child’s MVPA, we estimated the interaction effects of environmental attributes on the association between time and child’s MVPA (i.e., whether these environmental attributes were associated with change in child’s MVPA). The GAMMs explaining the association between time and children’s MVPA included a dichotomous time variable as a covariate and an additional random intercept variance component (within-person level) because these MVPA outcomes were measured twice (two years apart) for all included participants. We also examined the three-way interaction effects of environmental attributes by child’s age by time on child’s MVPA and environmental attributes by child’s sex by time on child’s MVPA. All analyses were conducted in R version 4.0.2 [52] using the packages ‘mgcv’ version 1.8.33 [54] and ‘multcomp’ version 1.4.13 s[55]. All analyses were performed initially with complete cases only (results are presented in Supplementary file 3), and then using multiple imputations.

## Results

The sample comprised 727 child-parent dyads whose demographics are presented in Table 1. Almost half (47%) resided in San Diego, the remainder (53%) in King County/Seattle. Most of the participating parents were mothers or female caregivers (86%). The mean age of parents was 41.6 (SD 5.8) years and almost all (94%) were married or in a partnered relationship. Overall, 39% of parents had completed college, a further 37% had completed a higher tertiary qualification, and 63% were employed. The median number of children per household was 2 (interquartile range or IQR 1) and the mean length of residence in the neighborhood was 100 (SD 61) months. The median number of motor vehicles was 2 (range 0–10), and the median number of adults with driver’s licenses was 2 (range 0–5) per household. The mean age of children in our study was 9.1 (SD 1.6) years at T1; there was even distribution by sex, and 16% were Hispanic. Children accumulated an average of 146 min/day (SD 53) of MVPA at T1, and 113 (SD 58) min/day at T2.

**Table 1:** Sample characteristics and environmental attributes (*N* = 727)

	**Overall (*n* = 727) **M ± SD or %	**San Diego (*n* = 345) **M ± SD or %	**Seattle (*n* = 382) **M ± SD or %
**Household characteristics**			
Parent’s age (years) *[4.8% missing]*	41.58 ± 5.81	41.46 ± 6.19	41.68 ± 5.47
Parent’s sex (% female) *[0% missing]*	86.52	86.67	86.39
Child’s age (years) *[0% missing]*	9.06 ± 1.56	9.23 ± 1.60	8.90 ± 1.52
Child’s sex (% female) *[0% missing]*	50.21	50.14	50.26
Child ethnicity (% Hispanic) *[4.4% missing]*	15.97	27.33	6.17
Highest Parental Education (%) *[5.2% missing]*			
Up to some college	23.51	33.12	15.18
Completed college	39.48	33.13	44.99
More than college degree	37.01	33.75	39.84
Employment (% working outside home) *[6.1% missing]*	62.52	64.44	60.87
Marital status (% Married/partnered) *[5.1% missing]*	93.91	91.22	96.23
Number of children *[0% missing]*	2.35 ± 0.89	2.26 ± 0.91	2.42 ± 0.86
Number of vehicles *[5.4% missing]*	2.44 ± 0.99	2.47 ± 1.07	2.41 ± 0.91
Number of adults with driver’s licenses *[5.5% missing]*	2.09 ± 0.52	2.07 ± 0.55	2.11 ± 0.48
Time in neighborhood (months) *[5.0% missing]*	100.4 ± 60.74	99.77 ± 60.55	101.0 ± 60.99
Neighborhood self-selection *[4.1% missing]*			
transport-related PA	3.07 ± 0.81	3.04 ± 0.88	3.10 ± 0.75
leisure-time PA	3.54 ± 0.88	3.49 ± 0.94	3.59 ± 0.82
safety and SES	4.12 ± 0.79	4.10 ± 0.86	4.14 ± 0.72
SES (Census-based median household income in ‘000) *[0% missing]*	63.69 ± 22.22	56.72 ± 20.79	69.99 ± 21.60
**Environmental attributes**			
PA equipment at home *[4.5% missing]*	5.76 ± 1.47	5.61 ± 1.50	5.89 ± 1.43
Traffic (self-report) [1–4] *[4.7% missing]*	2.62 ± 0.51	2.62 ± 0.52	2.63 ± 0.49
Crime (self-report) [1–4] *[4.7% missing]*	2.93 ± 0.67	2.86 ± 0.70	3.00 ± 0.63
Land use mix (audit) [0–61] *[0.3 missing]*	2.48 ± 2.64	2.78 ± 2.85	2.21 ± 2.41
Positive AT score (audit) [0–77] *[0.3% missing]*	11.23 ± 3.80	11.74 ± 3.36	10.77 ± 4.11
Intersection density (GIS) [0–1] *[0% missing]*	0.32 ± 0.07	0.32 ± 0.07	0.31 ± 0.08
Residential density (audit) *[0.3% missing]*			
Commercial	0.14	0.29	0
Single family	75.03	65.80	83.42
Multi family	24.83	33.91	16.58
Park PA facilities score (audit) [2–42] *[0.3% missing]*	12.10 ± 12.11	13.15 ± 12.68	11.14 ± 11.50
*[0% missing]*			
Informal play space score (audit) [0–48]	7.10 ± 10.46	5.62 ± 9.29	8.43 ± 11.27
*[0% missing]*			
Parks (GIS)			
no. within block groups	1.95 ± 2.06	1.48 ± 1.42	2.38 ± 2.42
presence within block groups (% yes)	74.43	76.49	72.58
*[22.0% missing]*			
no. within 1 km buffer presence	1.27 ± 1.89	1.0 ± 1.36	1.52 ± 2.23
within 1 km buffer (% yes)	56.40	55.07	57.59
[0% missing]			
**Accelerometry data**			
MVPA (T1; average min/day)	145.81 ± 53.38	137.81 ± 54.25	152.73 ± 51.69
*[4.3% missing]*			
MVPA (T2; average min/day)	112.50 ± 57.52	98.35 ± 41.57	125.96 ± 66.71
*[16.5% missing]*			
Change in MVPA			
(average min/day; T1 – T2)	-33.70 ± 46.32	-40.11 ± 37.34	-27.77 ± 52.68
*[17.9% missing]*			

*M* Mean, *SD* Standard deviation, *SES* Socio-economic status, *PA* Physical activity, *SESMAPS* Microscale audit of pedestrian streets caps, *MVPA* Moderate to vigorous PA, *GIS* Geographic information system, Land use mix – measured using ‘MAPS Destination Land Use (DLU) positive overall’ score; Positive AT score – a MAPS subscale measuring positive characteristics of the neighborhood for promoting active transport (AT); Park PA facilities score—measured using the Environmental Assessment of Public Recreational Spaces (EAPRS) audit tool; Play space score was measured using Informal Play Space audit tool; Land use mix – measured using ‘MAPS Destination Land Use (DLU) positive overall’ score; Residential density – measured using ‘MAPS Res_Density_Mix_recode’ score

In Table 2 the results are presented of our examination of first the cross-sectional total effects and then the direct effects of environmental attributes on child’s MVPA at T1, using multiple imputations. No significant associations were found.

**Table 2:** Results of regression^a^ analyses: effects of environmental attributes on children’s physical activity at T1 (using multiple imputations)

	**Total effect**		**Direct effect**	
	***b* (95% CI)**	***p***	***b* (95% CI)**	***p***
Informal play space score	-0.03 (-0.30, 0.24)	0.844	-0.03 (-0.30, 0.24)	0.844
Positive AT score	0.44 (-0.38, 1.26)	0.296	0.38 (-0.49, 1.24)	0.394
Residential density (ref: single family)				
Multi-family	1.33 (-5.62, 8.29)	0.708	3.14 (-5.14, 11.42)	0.458
Commercial	-0.91 (-72.12, 70.30)	0.980	-8.40 (-0.84, 67.37)	0.828
Park PA facilities score	0.17 (-0.08, 0.41)	0.179	0.17 (-0.08, 0.41)	0.179
Land use mix	0.59 (-0.68, 1.85)	0.365	0.44 (-0.90, 1.79)	0.520
Intersection density	-2.49 (-47.34, 42.37)	0.914	-8.43 (-57.26, 40.39)	0.735
No. of parks within 1 km	1.10 (-0.64, 2.84)	0.215	1.10 (-0.64,2.84)	0.215
PA equipment at home	1.25 (-0.78, 3.28)	0.230	1.25 (-0.78, 3.28)	0.230
Traffic safety	-2.67 (-9.11, 3.77)	0.417	-2.67 (-9.11, 3.77)	0.417
Crime safety	3.34 (-1.62, 8.30)	0.188	3.34 (-1.62, 8.30)	0.188

^*a*^Generalised additive mixed model (GAMM) with Gaussian distribution for all environmental attributes on moderate to vigorous physical activity (MVPA) at T1; Total and direct effect models were adjusted for census block group cluster id and accelerometer non-wear time; direct effect models were also adjusted for covariates listed in Table S4-1 (Supplementary file 4);

^b^ = regression coefficient; CI = confidence interval; Play space score was measured using Informal Play Space audit tool; Positive AT score – a MAPS subscale measuring positive characteristics of the neighborhood for promoting active transport (AT); Park PA facilities score—measured using the Environmental Assessment of Public Recreational Spaces (EAPRS) audit tool; Land use mix – measured using ‘MAPS Destination Land Use (DLU) positive overall’ score; Residential density – measured using ‘MAPS Res_Density_Mix_recode’ score

Next the moderating effects of child’s sex and child’s age on the associations between environmental attributes and child’s MVPA from multiple imputations were examined. None of the two-way interaction effects of child’s age and sex on these associations were statistically significant (Table S4-2, Supplementary file 4).

Next, we explored the interaction effects of environmental attributes on the association between time and child’s MVPA to examine whether environmental attributes were related to the changes in child’s MVPA. The results are presented in Table 3. The interaction of informal play space score by time was significant for children’s change in MVPA from T1 to T2. When we examined the interaction effect of time by informal play space we found there was no significant change in MVPA if no informal play space was present. However, if the informal play space score was present and above average (i.e., higher quality, more amenities) then child’s MVPA at T2 tended to be higher. In contrast, higher residential density, higher scores for PA facilities in nearby parks, and higher land use mix were associated with greater declines in children’s MVPA from T1 to T2. For illustrative purposes, the marginal means of MVPA at T1 and T2 were computed and plotted for those with an informal play space score of 0, average and above average score (Figure S4-1, Supplementary file 4). This was repeated for residential density, PA facilities score and land use mix (Figure S4-1, Supplementary file 4).

**Table 3:** The interaction effects of environmental attributes on the association between time and child’s MVPA from multiple imputations

Effect estimated		Regression models^a^	
		***b* (95% CI) *p***	
Main effect of Time on MVPA	Main	**-33.91 (-38.04, -29.78)**	** < 0.001**
Moderating effect of child’s sex on the association between time and MVPA	Moderator: Sex	0.83 (-6.46, 8.12)	0.823
Interaction effects of Play space score on the association between time and MVPA	Interaction	**0.42 (0.07, 0.78)**	**0.021**
	@ 0	-0.09 (-0.37, 0.20)	0.555
	@ average	**2.92 (0.53, 5.30)**	**0.018**
	@ above average	**7.34 (1.25, 13.43)**	**0.019**
Interaction effects of Positive AT score on the association between time and MVPA	Interaction	-0.61 (-1.53, 0.32)	0.200
Interaction effects of Residential density on the association between time and MVPA	Interaction	**-10.59 (-18.63, -2.55)**	**0.010**
	@ Single family	**-31.29 (-35.73, -26.85)**	** < 0.001**
	@ Multi-family	**-41.88 (-49.43, -34.33)**	** < 0.001**
Interaction effects of Park PA facilities score on the association between time and MVPA	Interaction	**-0.49 (-0.79, -0.19)**	**0.001**
	@ 0	**0.33 (0.08, 0.58)**	**0.010**
	@ average	**-5.60 (-9.08, -2.12)**	**0.002**
	@ above average	**-11.54 (-18.61, -4.46)**	**0.002**
Interaction effects of Land use mix positive overall on the association between time and MVPA	Interaction	**-1.68 (-3.06, -0.31)**	**0.017**
	@ 0	0.99 (-0.35, 2.33)	0.146
	@ average	**-3.19 (-6.21, -0.17)**	**0.040**
	@ above average	**-7.64 (-14.18, -1.11)**	**0.023**
Interaction effects of intersection density on the association between time and MVPA	Interaction	-22.15 (-75.83, 31.52)	0.420
Interaction effects of number of parks in 1 km buffer on the association between time and MVPA	Interaction	-1.26 (-3.26, 0.74)	0.217
Interaction effects of PA equipment at home on the association between time and MVPA	Interaction	1.73 (-0.73, 4.18)	0.169
Interaction effects of perceived traffic on the association between time and MVPA	Interaction	0.33 (-6.60, 7.26)	0.926
Interaction effects of perceived crime on the association between time and MVPA	Interaction	2.04 (-3.34, 7.42)	0.459

Note. ^a^generalised additive mixed model (GAMM) with gaussian distribution used for child’s MVPA, adjusted for same covariates as for Direct effects. All models also adjusted for census block group cluster id, within-individual levels and accelerometer wear-time at both time-points; b = regression coefficient; CI = confidence interval; Play space score was measured using Informal Play Space audit tool; Positive AT score – a MAPS subscale measuring positive characteristics of the neighborhood for promoting active transport (AT); Park PA facilities score—measured using the Environmental Assessment of Public Recreational Spaces (EAPRS) audit tool; Land use mix – measured using ‘MAPS Destination Land Use (DLU) positive overall’ score

None of the three-way interaction effects of environmental attributes by child’s age by time and environmental attribute by child’s sex by time on MVPA were statistically significant (data not presented).

## Discussion

This study aimed to examine how home and neighborhood environments in San Diego, CA and Seattle, WA in the United States were associated with initial and change in children’s PA over two years, by focusing on environmental characteristics that are considered to be supportive of PA. On average children’s MVPA declined over two years, and this aligned with many studies demonstrating that children become less active as they grow older [4, 5, 56]. Child’s sex did not moderate the association between time and MVPA. This was surprising because there is evidence that on average boys have greater declines in MVPA than girls, yet remain more active overall than girls since they start from a higher level of PA [5].

No significant associations were identified between environmental attributes and children’s MVPA at baseline. This is consistent with evidence [28, 35] that neighborhood environmental factors may be less influential on children’s than adult’s physical activity, at least among the environmental factors examined herein. This is especially true for younger children who stay closer to home and do not experience as much of their surrounding environments as older youth [57] who are more independent, journey further, and visit stores, eateries, parks, and other destinations. Lack of significant correlates could also be related to our examination of overall MVPA rather than MVPA during specific time periods (e.g. after school, when children are likely to have more access to their neighborhoods) or when the outcome is a specific type or domain of physical activity (e.g., active transport) [16, 32]. For example, in previous analyses of NIK T1 data, children’s accelerometer-based PA outside of school hours and parent reports of children’s active travel and PA in the neighborhood were all significantly related to MAPS streetscape audit summary scores in contrast to present results with total MVPA [58]. From a public health perspective, we considered it important to identify which environmental attributes are associated with overall MVPA. There is already evidence that children and adolescents who spent more time playing outdoors and walking in their neighborhood are more active overall [59–61].

Several environmental characteristics did appear, however, to impact the degree of decline in children’s MVPA over two years. Most notably, informal play spaces close to home were identified as being important for potentially offsetting age-related declines in children’s MVPA. This concurs with findings from a study in Spain that reported an inverse association between distance to playgrounds and overall PA among 6–12 year-olds [62]. Having informal play spaces close to home is expected to promote unstructured active play that parents may encourage due to convenience, proximity and opportunities for discreet surveillance by parents (if the play space is visible from home) or by neighbors [29, 32]. Broader surveillance from neighboring homes via ‘eyes on the street’ plays an important role in promoting neighborhood safety [46]. It could also be that, two years later, parents were more willing for these children to be active in proximal informal play spaces independently [63] or in the company of friends and/or other children in the neighborhood.

The contrasting finding that more PA facilities in nearby parks were associated with greater declines in MVPA among children appears counterintuitive. However, it is possible that parents of children in this age-group do not consider parks as safe venues to play alone and feel a need to accompany their children there, resulting in less habitual, spontaneous play and less frequent visits compared with independently mobile children [64] and compared with likely more proximal informal play spaces within the neighborhood.

Higher residential density was also unexpectedly associated with greater decline in children’s MVPA. This could be due to proportionally less space to play per child if more land is occupied by housing, or it could be that dwelling density is a proxy for SES in urban areas and facilities may be of poorer quality in lower-SES areas [31]. Another possible interpretation is that single/detached family homes are more likely to have private or minimally shared yards, driveways, or alley/street space on which children can play, compared to apartment complexes or other higher density residential units or areas. Studies have shown that low density disconnected environments are places where cul-de-sacs become de-facto youth play spaces [45]. Similarly, greater land use mix with walkable destinations, which often co-occurs with higher residential density, was associated with greater declines in children’s MVPA in the present study. Whilst the concept of walkability remains one that is consistently related to adults’ PA [35], it is unclear why negative associations between land use mix and children’s PA were found in the current study. It could be that having only residential land uses near a child’s home promotes lesser declines in children’s PA through similar mechanisms by which lower residential density prevents greater declines – more peer/friends available and more places to play with peers/friends in informal communal or private settings (i.e., friends’ yards, driveways). It is noteworthy that by the two-year follow-up, nearly all of the children in the present study were not yet in high school, so their independent mobility was likely limited by concerned caregivers.

Strengths of this study include the device-based measure of children’s total MVPA at two time-points, two years apart, and the detailed objective measurement of environmental features within two regions. Analyses included the application of multiple imputation of missing data to increase the number of study participants with complete data. Our study differs from previous research in its examination of a more comprehensive multi-level model of a diverse range of environmental correlates, including PA equipment types at home and formal (parks) and informal (play spaces) resources in the neighborhood. These included objective data from audits of informal play spaces close to children’s homes, using a novel instrument, and audits of PA facilities within local parks, as well as parental perceptions of crime and traffic gathered at baseline. Conducting the study in two separate regions provides increased diversity of environments and generalizability; perhaps primarily to other similar west coast cities in the US.

It is important, however, to acknowledge some limitations of this study. In particular, the environmental measures were measured only once at baseline (T1) and our analyses did not account for any infrastructure changes during the study period. The present analyses were exploratory. Whereas NIK neighborhoods were by study design selected based on differences in built environment characteristics, selection was not based on all of the characteristics examined in the present analysis. For example, it could be that neighborhoods examined did not simultaneously have adequately supportive environments for walkability (higher residential density, even greater land use mix), parks with more physical activity facilities, and ample and adequate informal play spaces and/or that children were less likely to live in the more walkable areas of the regions studied. Present analyses were based on observational data and were performed to start to identify those environmental characteristics it might be important to consider in understanding changes in children’s physical activity in middle childhood. Interventions that modify environmental characteristics (and combinations thereof) and children’s exposure to them would provide more definitive evidence regarding environmental influences on changes in physical activity. Findings may not be generalizable to other populations of children nationally or internationally, or to youth of different ages, or to different contexts that have different levels of these environmental characteristics or different types altogether.

## Conclusions

Present exploratory findings highlight the potential importance of having high-quality informal play spaces close to children’s homes to encourage habitual, spontaneous play and help to stem age-related declines in PA. In contrast, present findings suggest that some factors consistently related to higher adults’ physical activity, particularly active transportation, are related to greater declines in youth physical activity in the examined age range. Future studies should determine whether present these findings can be replicated and evaluate interventions that construct or retrofit informal play spaces in residential areas and measure their impact on children’s MVPA levels.

## Supplementary Information

**Additional file** 1.

**Additional file2: Figure S2-1.** Directed Acyclic Graph (DAG) of proposed associations between environmental characteristics and children’s moderate-to-vigorous physical activity. This particular example shows the effect of neighborhood self-selection (relevant to leisure PA) on MVPA and its potential confounders (in red)

**Additional file 3: Table S3-1.** Estimation of total and direct effects of environmental attributes on child’s MVPA at T1. **Table S3-2.** Moderating effects of child’s sex and child’s age on the association between environmental attribute and child’s MVPA. **Table S3-3.** Interaction effects of environmental attributes on the association between time and child’s MVPA. 

**Additional file 4: Table S4-1.** Effects of environmental attributes on children’s physical activity at T1 (using multiple imputations). **Table S4-2.** Moderating effects of child’s sex and child’s age on the association between environmental attribute and child’s MVPA from multiple imputations. **Figure S4-1.** Marginal means for moderate-to-vigorous physical activity (MVPA)  at Times 1 and 2 where there were significant interaction effects of environmental attributes on the association between timeand child’s MVPA

## Data Availability

As the consent forms signed by participants indicated that the data would be only accessible to the team of investigators, the data are confidential.

## Abbreviations

AT: Active transportation
DAG: Directed acyclic graph
EAPRS: Environmental Assessment of Public Recreational Spaces
GAMMs: Generalized additive mixed models
GIS: Geographic information systems
IQR: Interquartile range
MAPS: Microscale Audit of Pedestrian Streetscapes
MAR: Missing at random
MCAR: Missing completely at random
MVPA: Moderate-to-vigorous physical activity
NEWS-Y: Neighborhood Environment Walkability Survey – Youth version
NIK: Neighborhood Impact on Kids
PA: Physical activity
SD: Standard deviation
SES: Socioeconomic status
T1: Time 1/baseline
T2: Time 2/two-year follow-up
