# Supplementary material for: Associations of home and neighborhood environments with children’s physical activity in the U.S.-based Neighborhood Impact on Kids (NIK) longitudinal cohort study
Source: Int J Behav Nutr Phys Act. 2023 Feb 2;20:9. doi: 10.1186/s12966-023-01415-3 (PMC9896701; doi:10.1186/s12966-023-01415-3)
Supplement: Supplementary file 1 — Additional file 1. [file 12966_2023_1415_MOESM1_ESM.doc]

**NIK Informal Play Space Evaluation Entry 1: Rater______ Date Entered:________**

Version 1.2

**Date Auditor ID#**  **Entry 2: Rater______ Date Entered:________**

**ID#**

**Type: ; Size: sq. ft.**

1. Is the play space part of an apartment or condominium complex? ⁪1 Yes ⁪0 No

1a. If yes, what is the name of the complex? __________________________________________________

2. Is the play space part of a planned housing development/HOA? ⁪1 Yes ⁪0 No

2a. If yes, what is the name of the development? _______________________________________________

3. What is the address or cross-streets of the play space? ____________________________________________________________

4. What is the name of the play space (if applicable)? ____________________________________________________________

5. Quality of walking connection between NIK child residence and play space

⁪1 Poor ⁪2 Fair ⁪3 Excellent ⁪-777 N/A No path needed

(e.g., adjacent)

6. Distance to informal play space ⁪1 <1000 feet ⁪2 1000-2000 feet ⁪3 >2000 feet

7. Surface/ground condition ⁪1 Poor ⁪2 Fair ⁪3 Excellent

8. Flatness of the play space ⁪1 Significant grade ⁪2 Some grade ⁪3 Mostly or all flat

9. Are there both paved and unpaved play ⁪1 Yes ⁪0 No

areas available (>500 square feet each

not including any paths or trails, but can include courts as paved)

10. Amenities/facilities available

a. Trail (>200 feet long) ⁪1 Yes ⁪0 No

b. Open space (>500 square feet; unpaved) ⁪1 Yes ⁪0 No

c. Pond, lake, or stream ⁪1 Yes ⁪0 No

d. Swimming or wading pool (whether open or not) ⁪1 Yes ⁪0 No

e. Restroom (open) ⁪1 Yes ⁪0 No

f. Playset (≥2 different attached play equipment) ⁪1 Yes ⁪0 No

g. Swings (not part of playset) ⁪1 Yes ⁪0 No

h. Slides (not part of playset) ⁪1 Yes ⁪0 No

i. Things to climb or hang from (not part of playset) ⁪1 Yes ⁪0 No

j. Basketball hoop ⁪1 Yes ⁪0 No

k. Athletic field ⁪1 Yes ⁪0 No

l. Athletic court (lined; non-basketball) ⁪1 Yes ⁪0 No

m. Other ___________________________ ⁪1 Yes ⁪0 No

11. Amenities/facilities condition ⁪1 Poor ⁪2 Fair ⁪3 Excellent ⁪-777 N/A – no

amenities

12. Overall cleanliness ⁪1 Not at all ⁪2 Somewhat ⁪3 Mostly to extremely

13. Size of play space ⁪1 500 – 1500 sq ft ⁪2 1501-2000 sq ft ⁪3 >2000 sq ft

*(calculated by walking the perimeter of at least 2 sides)*

14. Barrier between play space and road ⁪1 <25% ⁪2 26-50% ⁪3 51-75% ⁪4 76-100%

15. Visibility from NIK child residence ⁪1 Poor ⁪2 Fair ⁪3 Excellent

16. Visibility from other residences ⁪1 Poor ⁪2 Fair ⁪3 Excellent

17. Any signage discouraging active play or other active behavior (e.g., skateboarding) ⁪1 Yes ⁪0 No

18. Any lighting ⁪1 Yes ⁪0 No

NIK – Neighborhood Impact on Kids study
