# Supplementary material for: Associations of home and neighborhood environments with children’s physical activity in the U.S.-based Neighborhood Impact on Kids (NIK) longitudinal cohort study
Source: Int J Behav Nutr Phys Act. 2023 Feb 2;20:9. doi: 10.1186/s12966-023-01415-3 (PMC9896701; doi:10.1186/s12966-023-01415-3)
Supplement: Supplementary file 2 — Additional file2: Figure S2-1. Directed Acyclic Graph (DAG) of proposed associations between environmental characteristics and children’s moderate-to-vigorous physical activity. This particular example shows the effect of neighborhood self-selection (relevant to leisure PA) on MVPA and its potential confounders (in red) [file 12966_2023_1415_MOESM2_ESM.docx]

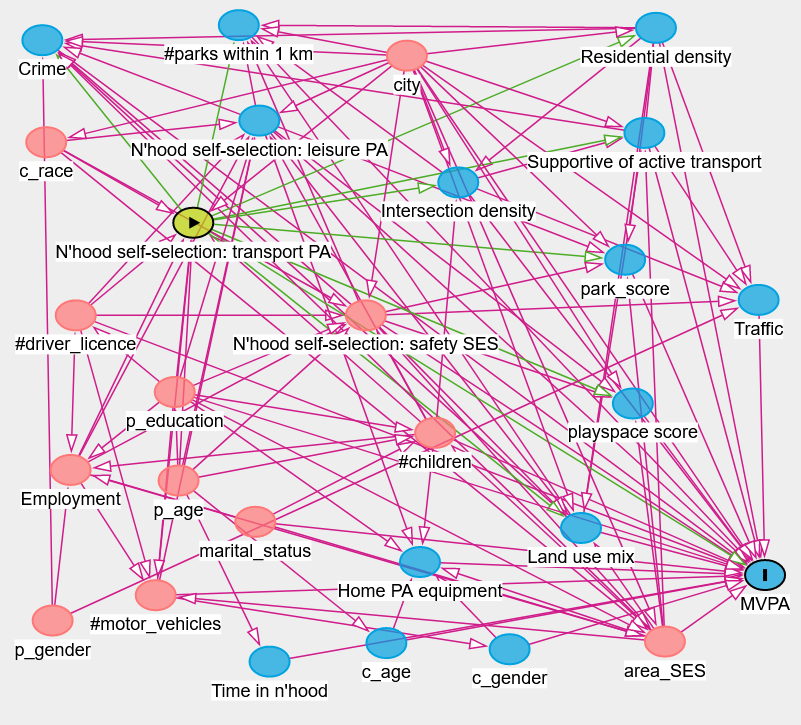


**Figure S2-1. Directed Acyclic Graph (DAG) of proposed associations between environmental characteristics and children’s moderate-to-vigorous physical activity. This particular example shows the effect of neighborhood self-selection (relevant to leisure PA) on MVPA and its potential confounders (in red)**

**Legend**


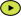
 exposure


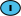
 outcome


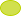
 ancestor of exposure


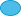
 ancestor of outcome

 ancestor of exposure and outcome


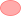


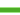
 causal path


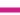
 biasing path

**Variable names with prefix ‘p_’ denote characteristics of parents, and those with prefix ‘c_’ denote characteristics of children.**

**SES – socioeconomic status**

**N’hood – neighbourhood**

**PA – physical activity**

**MVPA – moderate -to-vigorous physical activity**

**# - number of**

**Corresponding R code to generate Figure S1:**

dag {

bb="-6.12,-5.835,5.542,7.806"

"#children" [pos="0.216,2.309"]

"#driver_licence" [pos="-5.017,0.098"]

"#motor_vehicles" [pos="-3.853,5.364"]

"#parks within 1 km" [pos="-2.643,-5.362"]

"Home PA equipment" [pos="-0.005,4.739"]

"Intersection density" [pos="0.552,-2.403"]

"Land use mix" [pos="2.339,4.099"]

"N'hood self-selection: leisure PA " [pos="-2.345,-3.566"]

"N'hood self-selection: safety SES" [pos="-0.794,0.098"]

"N'hood self-selection: transport PA " [exposure,pos="-3.306,-1.644"]

"Residential density" [pos="3.430,-5.311"]

"Supportive of active transport" [pos="3.262,-3.334"]

"Time in n'hood" [pos="-2.195,6.614"]

"playspace score" [pos="3.094,1.756"]

Crime [pos="-5.503,-5.079"]

Employment [pos="-5.092,3.007"]

MVPA [outcome,pos="5.019,4.985"]

Traffic [pos="4.925,-0.192"]

area_SES [pos="3.561,6.235"]

c_age [pos="-0.495,6.264"]

c_gender [pos="1.300,6.381"]

c_race [pos="-5.447,-3.159"]

city [pos="-0.196,-4.788"]

marital_status [pos="-2.403,3.983"]

p_age [pos="-3.520,3.206"]

p_education [pos="-3.576,1.541"]

p_gender [pos="-5.354,5.840"]

park_score [pos="2.982,-0.949"]

"#children" -> "Home PA equipment"

"#children" -> "Intersection density"

"#children" -> "N'hood self-selection: leisure PA "

"#children" -> "N'hood self-selection: safety SES"

"#children" -> "N'hood self-selection: transport PA "

"#children" -> Employment

"#children" -> MVPA

"#driver_licence" -> "#motor_vehicles"

"#driver_licence" -> "N'hood self-selection: leisure PA "

"#driver_licence" -> "N'hood self-selection: safety SES"

"#driver_licence" -> "N'hood self-selection: transport PA "

"#driver_licence" -> MVPA

"#driver_licence" <-> Employment

"#motor_vehicles" -> "N'hood self-selection: leisure PA "

"#motor_vehicles" -> "N'hood self-selection: transport PA "

"#motor_vehicles" -> MVPA

"#motor_vehicles" -> c_gender

"#parks within 1 km" -> MVPA

"Home PA equipment" -> MVPA

"Intersection density" -> "#parks within 1 km"

"Intersection density" -> "Supportive of active transport"

"Intersection density" -> MVPA

"Intersection density" -> Traffic

"Land use mix" -> "#parks within 1 km"

"Land use mix" -> "Supportive of active transport"

"Land use mix" -> Crime

"Land use mix" -> MVPA

"N'hood self-selection: leisure PA " -> "#parks within 1 km"

"N'hood self-selection: leisure PA " -> "Home PA equipment"

"N'hood self-selection: leisure PA " -> "Land use mix"

"N'hood self-selection: leisure PA " -> "playspace score"

"N'hood self-selection: leisure PA " -> Crime

"N'hood self-selection: leisure PA " -> MVPA

"N'hood self-selection: leisure PA " -> park_score

"N'hood self-selection: safety SES" -> "#parks within 1 km"

"N'hood self-selection: safety SES" -> "Land use mix"

"N'hood self-selection: safety SES" -> "playspace score"

"N'hood self-selection: safety SES" -> Crime

"N'hood self-selection: safety SES" -> MVPA

"N'hood self-selection: safety SES" -> Traffic

"N'hood self-selection: safety SES" -> area_SES

"N'hood self-selection: safety SES" -> park_score

"N'hood self-selection: transport PA " -> "#parks within 1 km"

"N'hood self-selection: transport PA " -> "Intersection density"

"N'hood self-selection: transport PA " -> "Land use mix"

"N'hood self-selection: transport PA " -> "Residential density"

"N'hood self-selection: transport PA " -> "Supportive of active transport"

"N'hood self-selection: transport PA " -> "playspace score"

"N'hood self-selection: transport PA " -> Crime

"N'hood self-selection: transport PA " -> MVPA

"N'hood self-selection: transport PA " -> park_score

"Residential density" -> "#parks within 1 km"

"Residential density" -> "Intersection density"

"Residential density" -> "Land use mix"

"Residential density" -> Crime

"Residential density" -> MVPA

"Residential density" -> Traffic

"Residential density" -> park_score

"Supportive of active transport" -> "Residential density"

"Supportive of active transport" -> Crime

"Supportive of active transport" -> MVPA

"Supportive of active transport" -> Traffic

"Time in n'hood" -> MVPA

"playspace score" -> MVPA

Crime -> MVPA

Employment -> "#motor_vehicles"

Employment -> "N'hood self-selection: leisure PA "

Employment -> "N'hood self-selection: safety SES"

Employment -> "N'hood self-selection: transport PA "

Employment -> area_SES

Traffic -> MVPA

area_SES -> "#children"

area_SES -> "#motor_vehicles"

area_SES -> "Home PA equipment"

area_SES -> "Land use mix"

area_SES -> "Supportive of active transport"

area_SES -> "playspace score"

area_SES -> Crime

area_SES -> MVPA

area_SES -> park_score

c_age -> "Home PA equipment"

c_age -> MVPA

c_gender -> "Home PA equipment"

c_gender -> MVPA

c_race -> "N'hood self-selection: leisure PA "

c_race -> "N'hood self-selection: safety SES"

c_race -> "N'hood self-selection: transport PA "

c_race -> area_SES

city -> "#parks within 1 km"

city -> "Intersection density"

city -> "Land use mix"

city -> "N'hood self-selection: leisure PA "

city -> "N'hood self-selection: safety SES"

city -> "N'hood self-selection: transport PA "

city -> "Residential density"

city -> "Supportive of active transport"

city -> "playspace score"

city -> Crime

city -> MVPA

city -> Traffic

city -> area_SES

city -> c_race

city -> park_score

marital_status -> "#children"

marital_status -> Employment

marital_status -> MVPA

marital_status -> area_SES

p_age -> "#children"

p_age -> "N'hood self-selection: leisure PA "

p_age -> "N'hood self-selection: safety SES"

p_age -> "N'hood self-selection: transport PA "

p_age -> "Time in n'hood"

p_age -> c_age

p_age -> p_education

p_education -> "#children"

p_education -> "#driver_licence"

p_education -> "#motor_vehicles"

p_education -> "Home PA equipment"

p_education -> "N'hood self-selection: leisure PA "

p_education -> "N'hood self-selection: safety SES"

p_education -> "N'hood self-selection: transport PA "

p_education -> Employment

p_education -> MVPA

p_education -> area_SES

p_gender -> Crime

p_gender -> Employment

p_gender -> Traffic

park_score -> MVPA

}
