# Supplementary material for: Associations of home and neighborhood environments with children’s physical activity in the U.S.-based Neighborhood Impact on Kids (NIK) longitudinal cohort study
Source: Int J Behav Nutr Phys Act. 2023 Feb 2;20:9. doi: 10.1186/s12966-023-01415-3 (PMC9896701; doi:10.1186/s12966-023-01415-3)
Supplement: Supplementary file 3 — Additional file 3: Table S3-1. Estimation of total and direct effects of environmental attributes on child’s MVPA at T1. Table S3-2. Moderating effects of child’s sex and child’s age on the association between environmental attribute and child’s MVPA. Table S3-3. Interaction effects of environmental attributes on the association between time and child’s MVPA. [file 12966_2023_1415_MOESM3_ESM.docx]

**Supplementary file 3 – Results from complete case analyses**

**Table S3-1. Estimation of total and direct effects of environmental attributes on child’s MVPA at T1**

| **Effect estimated** | **Covariates** | **Regression models^a^** | |  |
| --- | --- | --- | --- | --- |
|  |  | ***b* (95% CI) p** | |  |
| Total effects of Play space score on MVPA | Neighbourhood self-selection (3 scores), area_SES, child age, child sex, city | -0.03 (-0.30, 0.25) | 0.839 |  |
| Direct effects of Play space score on MVPA | Neighbourhood self-selection (3 scores), SES, child age, child sex, city | -0.03 (-0.30, 0.25) | 0.839 |  |
|  |  |  |  |  |
| Total effects of Positive AT score on MVPA | Land use mix, Int_density, Neighbourhood self-selection for transportation PA, SES, child age, child sex, city | 0.41 (-0.44, 1.25) | 0.344 |  |
| Direct effects of Positive AT score on MVPA | No. of children, No. of driver_licence, No. of motor_vehicles, Crime, Land use mix, Residential_density, Int_density, Neighbourhood self-selection (3 scores), Time in n'hood, Traffic, SES, child age, child sex, city, marital_status, parent’s educ | 0.41 (-0.47, 1.30) | 0.360 |  |
|  |  |  |  |  |
| Total effects of Residential density on MVPA (reference: Single-family)  Multi-family | Neighbourhood self-selection for transportation PA, child age, child sex, city | 1.42 (-5.48, 8.32) | 0.687 |  |
| Direct effects of Residential density on MVPA (reference: Single-family)  Multi-family | No. of children, No. of driver_licence, No. of motor_vehicles, No.of parks within 1 km, Crime, Land use mix, Int_density, MAPS_POS_AT, Neighbourhood self-selection (3 scores), Time in n'hood, Traffic, area_SES, child age, child sex, city, marital_status, parents educ, park_score | 3.25 (-5.22, 11.72) | 0.451 |  |
|  |  |  |  |  |
| Total effects of Park PA facilities score on MVPA | Residential_density, Neighbourhood self-selection (3 scores), SES, child age, child sex, city | 0.19 (-0.06, 0.44) | 0.135 |  |
| Direct effects of Park PA facilities score on MVPA | Residential_density, Neighbourhood self-selection (3 scores), SES, child age, child sex, city | 0.19 (-0.06, 0.44) | 0.135 |  |
|  |  |  |  |  |
| Total effects of Land use mix on MVPA | Residential_density, Neighbourhood self-selection (3 scores), child age, child sex, city | 0.61 (-0.66, 1.88) | 0.342 |  |
| Direct effects of Land use mix on MVPA | No. of children, No. of driver_licence, No. of motor_vehicles, No. of parks within 1 km, Crime, Residential_density, Int_density, MAPS_POS_AT, Neighbourhood self-selection (3 scores), Time in n'hood, Traffic, SES, child age, child sex, city, marital_status, parent’s educ | 0.47 (-0.88, 1.82) | 0.491 |  |
|  |  |  |  |  |
| Total effects of intersection density on MVPA | Residential_density, Neighbourhood self-selection for transportation PA, child age, child sex, city | -2.80 (-44.65, 39.05) | 0.895 |  |
| Direct effects of intersection density on MVPA | No. of children, No. of driver_licence, No. of motor_vehicles, No. of parks within 1 km, Crime, Land use mix, Residential_density, MAPS_POS_AT, Neighbourhood self-selection (3 scores), Time in n'hood, Traffic, SES, child age, child sex, city, marital_status, parent’s educ | -13.39 (-58.72, 31.94) | 0.562 |  |
|  |  |  |  |  |
| Total effects of number of parks in 1 km buffer on MVPA | Land use mix, Residential_density, Int_density, Neighbourhood self-selection (3 scores), child age, child sex, city | 1.20 (-0.54,2.95) | 0.177 |  |
| Direct effects of number of parks in 1 km buffer on MVPA | Land use mix, Residential_density, Int_density, Neighbourhood self-selection (3 scores), child age, child sex, city | 1.20 (-0.54,2.95) | 0.177 |  |

*a -* GAMM, generalised additive mixed model; all models were adjusted for clustering at the Census block group level cluster id and wear-time; b = regression coefficient; CI = confidence interval. Play space score was measured using Informal Play Space audit tool; Positive AT score – a MAPS subscale measuring positive characteristics of the neighbourhood for promoting active transport (AT); Park PA facilities score - measured using the Environmental Assessment of Public Recreational Spaces (EAPRS) audit tool; Land use mix – measured using ‘MAPS Destination Land Use (DLU) positive overall’ score; Int_density – intersection density; Residential density – measured using ‘MAPS Res_Density_Mix_recode’ score. Neighbourhood self-selection – 3 scores regarding transport-related PA, leisure-time PA, safety/SES, respectively.

**Table S3-2. Moderating effects of child’s sex and child’s age on the association between environmental attribute and child’s MVPA**

| **Effect estimated** | **Moderator** | **Regression models^a^** | |  |
| --- | --- | --- | --- | --- |
|  |  | ***b* (95% CI) p** | |  |
| Interaction effects of child’s sex and child’s age on the association between Play space score and MVPA | Sex  Age | 0.10 (-0.43, 0.63)  -0.04 (-0.22 0.13) | 0.709  0.649 |  |
|  |  |  |  |  |
| Interaction effects of child’s sex and child’s age on the association between Positive AT score and MVPA | Sex  Age | -0.07 (-1.59, 1.45)  0.19 (-0.28, 0.67) | 0.928  0.424 |  |
|  |  |  |  |  |
| Interaction effects of child’s sex and child’s age on the association between Residential density and MVPA | Sex  Age | -8.94 (-22.24, 4.36)  -3.11 (-7.49, 1.26) | 0.187  0.162 |  |
|  |  |  |  |  |
| Interaction effects of child’s sex and child’s age on the association between Park PA facilities score and MVPA | Sex  Age | -0.23 (-0.70, 0.23)  0.12 (-0.02, 0.27) | 0.329  0.096 |  |
|  |  |  |  |  |
| Interaction effects of child’s sex and child’s age on the association between Land use mix positive overall and MVPA | Sex  Age | -1.28 (-3.45, 0.88)  0.26 (-0.48, 1.00) | 0.245  0.490 |  |
|  |  |  |  |  |
| Interaction effects of child’s sex and child’s age on the association between intersection density and MVPA | Sex  Age | 10.22 (-71.41, 91.85)  8.06 (-17.65, 33.76) | 0.806  0.538 |  |
|  |  |  |  |  |
| Interaction effects of child’s sex and child’s age on the association between number of parks in 1 km buffer and MVPA | Sex  Age | -1.03 (-4.07, 2.01)  -0.08 (-1.02, 0.87) | 0.505  0.872 |  |

a - GAMM with gaussian distribution used for child’s MVPA, adjusted for same covariates as used for Direct effects. All models also adjusted for census block group cluster id and accelerometer wear-time. b = regression coefficient; CI = confidence interval. Play space score was measured using Informal Play Space audit tool; Positive AT score – a MAPS subscale measuring positive characteristics of the neighbourhood for promoting active transport (AT); Park PA facilities score - measured using the Environmental Assessment of Public Recreational Spaces (EAPRS) audit tool; Land use mix – measured using ‘MAPS Destination Land Use (DLU) positive overall’ score; Residential density – measured using ‘MAPS Res_Density_Mix_recode’ score.

None of the two-way interaction effects of child’s age and sex on the association between environmental attributes and MVPA were statistically significant.

**Table S3-3. Interaction effects of environmental attributes on the association between time and child’s MVPA**

| **Effect estimated** |  | **Regression models^a^** | |  |
| --- | --- | --- | --- | --- |
| **Effects** |  | ***b* (95% CI) p** | |  |
| Interaction effects of Play space on the association between time and MVPA | Interaction  @ 0  @ average | **0.51 (0.14, 0.87)**  -0.14 (-0.45, 0.18)  **3.46 (1.07, 5.84)** | **0.006**  0.404  **0.004** |  |
|  | @ above average | **8.76 (2.62,14.89)** | **0.005** |  |
| Interaction effects of Positive AT score on the association between time and MVPA | Interaction | -0.74 (-1.76, 0.27) | 0.150 |  |
|  |  |  |  |  |
| Interaction effects of Residential density on the association between time and MVPA | Interaction  @ Single family  @ Multi-family | **-11.97 (-20.98, -2.96)**  **-30.78 (-35.07, -26.49)**  **-42.75 (-50.54, -34.96)** | **0.009**  **<0.001**  **<0.001** |  |
|  |  |  |  |  |
| Interaction effects of Park PA facilities score on the association between time and MVPA | Interaction  @ 0  @ average  @ above average | **-0.55 (-0.85, -0.24)**  **0.38 (0.10, 0.66)**  **-6.22 (-9.76, -2.69)**  **-12.84 (-20.06, -5.61)** | **<0.001**  **0.007**  **<0.001**  **<0.001** |  |
|  |  |  |  |  |
| Interaction effects of Land use mix on the association between time and MVPA | Interaction  @ 0  @ average  @ above average | **-2.10 (-3.63, -0.57)**  1.35 (-0.15, 2.85)  **-3.86 (-7.11, -0.60)**  **-9.40 (-16.48, -2.32)** | **0.007**  0.078  **0.020**  **0.009** |  |
|  |  |  |  |  |
| Interaction effects of Intersection density on the association between time and MVPA | Interaction | -27.82 (-81.61, 25.98) | 0.310 |  |
|  |  |  |  |  |
| Interaction effects of number of parks in 1km residential buffers on the association between time and MVPA | Interaction | -1.99 (-4.09, 0.11) | 0.063 |  |

a - generalised additive mixed model (GAMM) with gaussian distribution used for child’s MVPA, adjusted for same covariates as used for direct effects. All models also adjusted for census block group cluster id, within-individual levels, accelerometer wear-time at T1 and T2; b = regression coefficient; CI = confidence interval. Play space score was measured using Informal Play Space audit tool; Positive AT score – a MAPS subscale measuring positive characteristics of the neighbourhood for promoting active transport (AT); Park PA facilities score - measured using the Environmental Assessment of Public Recreational Spaces (EAPRS) audit tool; Land use mix – measured using ‘MAPS Destination Land Use (DLU) positive overall’ score; Residential density – measured using ‘MAPS Res_Density_Mix_recode’ score.
