# Supplementary material for: Associations of home and neighborhood environments with children’s physical activity in the U.S.-based Neighborhood Impact on Kids (NIK) longitudinal cohort study
Source: Int J Behav Nutr Phys Act. 2023 Feb 2;20:9. doi: 10.1186/s12966-023-01415-3 (PMC9896701; doi:10.1186/s12966-023-01415-3)
Supplement: Supplementary file 4 — Additional file 4: Table S4-1. Effects of environmental attributes on children’s physical activity at T1 (using multiple imputations). Table S4-2. Moderating effects of child’s sex and child’s age on the association between environmental attribute and child’s MVPA from multiple imputations. Figure S4-1. Marginal means for moderate-to-vigorous physical activity (MVPA) at Times 1 and 2 where there were significant interaction effects of environmental attributes on the association between timeand child’s MVPA [file 12966_2023_1415_MOESM4_ESM.docx]

**Table S4-1. Effects of environmental attributes on children’s physical activity at T1 (using multiple imputations)**

| **Effect estimated** | **Covariates** | **Regression model^a^** | |
| --- | --- | --- | --- |
|  |  | ***b* (95% CI) p** | |
| Total effects of Play space score on MVPA | Neighbourhood self-selection (3 scores), SES, child age, child sex, city | -0.03 (-0.30, 0.24) | 0.844 |
| Direct effects of Play space score on MVPA | Neighbourhood self-selection (3 scores), SES, child age, child sex, city | -0.03 (-0.30, 0.24) | 0.844 |
|  |  |  |  |
| Total effects of Positive AT score on MVPA | Land use mix, Int_density, Neighbourhood self-selection for transportation PA, SES, child age, child gender, city | 0.44 (-0.38, 1.26) | 0.296 |
| Direct effects of Positive AT score on MVPA | No. of children, No. of driver_licence, No. of motor_vehicles, Crime, Land use mix, Residential_density, Int_density, Neighbourhood self-selection (3 scores), Time in n'hood, Traffic, SES, child age, child sex, city, marital_status, Parent’s education | 0.38 (-0.49, 1.24) | 0.394 |
|  |  |  |  |
| Total effects of Residential density on MVPA (reference: Single family)  Multi-family  Commercial | Neighbourhood self-selection for transportation PA, child age, child sex, city | 1.33 (-5.62, 8.29)  -0.91 (-72.12, 70.30) | 0.708  0.980 |
| Direct effects of Residential density on MVPA (reference: Single-family)  Multi-family  Commercial | No. of children, No. of driver_licence, No. of motor_vehicles, No. of parks within 1 km, Crime, Land use mix, Int_density, Positive AT score, Neighbourhood self-selection (3 scores), Time in n'hood, Traffic, SES, child age, child sex, city, marital_status, parent’s_education, park_score | 3.14 (-5.14, 11.42)  -8.40 (-0.84, 67.37) | 0.458  0.828 |
|  |  |  |  |
| Total effects of Park PA facilities score on MVPA | Residential_density, Neighbourhood self-selection (3 scores), SES, child age, child sex, city | 0.17 (-0.08, 0.41) | 0.179 |
| Direct effects of Park PA facilities score on MVPA | Residential_density, Neighbourhood self-selection (3 scores), SES, child age, child sex, city | 0.17 (-0.08, 0.41) | 0.179 |
|  |  |  |  |
| Total effects of Land use mix on MVPA | Residential_density, Neighbourhood self-selection (3 scores), child age, child sex, city | 0.59 (-0.68, 1.85) | 0.365 |
| Direct effects of Land use mix on MVPA | No. of children, No. of driver_licence, No. of motor_vehicles, No. of parks within 1 km, Crime, Residential_density, Int_density, Positive AT score, Neighbourhood self-selection (3 scores), Time in n'hood, Traffic, SES, child age, child sex, city, marital_status, parent’s education | 0.44 (-0.90, 1.79) | 0.520 |
|  |  |  |  |
| Total effects of intersection density on MVPA | Residential_density, Neighbourhood self-selection for transportation PA, child age, child sex, city | -2.49 (-47.34, 42.37) | 0.914 |
| Direct effects of intersection density on MVPA | No. of children, No. of driver_licence, No. of motor_vehicles, No. of parks within 1 km, Crime, Land use mix, Residential_density, Positive AT score, Neighbourhood self-selection (3 scores), Time in n'hood, Traffic, SES, child age, child sex, city, marital_status, parent’s education | -8.43 (-57.26, 40.39) | 0.735 |
|  |  |  |  |
| Total effects of number of parks in 1 km buffer on MVPA | Land use mix, Residential_density, Int_density, Neighbourhood self-selection (3 scores), age, gender, city | 1.10 (-0.64, 2.84) | 0.215 |
| Direct effects of number of parks in 1 km buffer on MVPA | Land use mix, Neighbourhood self-selection (3 scores), child age, child sex, Residential_density, Int_density, city | 1.10 (-0.64,2.84) | 0.215 |
| Total effects of PA equipment at home on MVPA | No. of children, NSS_LPA, SES, child age, child sex, parent’s education | 1.25 (-0.78, 3.28) | 0.230 |
| Direct effects of PA equipment at home on MVPA | No. of children, NSS_LPA, SES, child age, child sex, parent’s education | 1.25 (-0.78, 3.28) | 0.230 |
| Total effects of perceived traffic on MVPA | No. of children, No. of driver licence, No. of motor vehicles, Crime, Land use mix, Residential_density, Int_density, Positive AT score, Neighbourhood self-selection (3 scores), Time in n'hood, SES, child age, city, marital_status, parent’s education | -2.67 (-9.11, 3.77) | 0.417 |
| Direct effects of perceived traffic on MVPA | Same as above | -2.67 (-9.11, 3.77) | 0.417 |
| Total effects of perceived crime on MVPA | No. of children, No. of driver licence, No. of motor vehicles, Land use mix, Residential_density, Int_density, Positive AT score, Neighbourhood self-selection (3 scores), Time in n'hood, Traffic, SES, child age, city, marital status, parent’s education | 3.34 (-1.62, 8.30) | 0.188 |
| Direct effects of perceived crime on MVPA | Same as above | 3.34 (-1.62, 8.30) | 0.188 |

*a*  Generalised additive mixed model (GAMM) with gaussian distribution for all environmental attributes on moderate to vigorous physical activity (MVPA) at T1; adjusted for census block cluster id and accelerometer wear-time; b = regression coefficient; CI = confidence interval.

Play space score was measured using Informal Play Space audit tool; Positive AT score – a MAPS subscale measuring positive characteristics of the neighbourhood for promoting active transport (AT); Residential density – measured using ‘MAPS Res_Density_Mix_recode’ score; Park PA facilities score - measured using the Environmental Assessment of Public Recreational Spaces (EAPRS) audit tool; Land use mix – measured using ‘MAPS Destination Land Use (DLU) positive overall’ score ; Int_density – intersection density; Neighbourhood self-selection (3 scores),: neighbourhood self-selection sores related to leisure PA, safety and socioeconomic status, and transportation PA, respectively.

**Table S4-2. Moderating effects of child’s sex and child’s age on the association between environmental attribute and child’s MVPA from multiple imputations**

| **Effect estimated** |  | **Regression models^a^** | |
| --- | --- | --- | --- |
|  |  | ***b* (95% CI) p** | |
| Interaction effects of child’s sex and child’s age on the association between Play space score and MVPA | Sex  Age | 0.12 (-0.41, 0.65)  -0.02 (-0.20 0.16) | 0.652  0.796 |
|  |  |  |  |
| Interaction effects of child’s sex and child’s age on the association between Positive AT score and MVPA | Sex  Age | -0.18 (-1.66, 1.30)  0.13 (-0.33, 0.59) | 0.810  0.578 |
|  |  |  |  |
| Interaction effects of child’s sex and child’s age on the association between Residential density and MVPA | Sex  Age | -8.81 (-21.64, 4.02)  -2.54 (-6.88, 1.80) | 0.179  0.251 |
|  |  |  |  |
| Interaction effects of child’s sex and child’s age on the association between Park PA facilities score and MVPA | Sex  Age | -0.25 (-0.71, 0.21)  0.11 (-0.03, 0.25) | 0.291  0.126 |
|  |  |  |  |
| Interaction effects of child’s sex and child’s age on the association between Land use mix and MVPA | Sex  Age | -1.34 (-3.47, 0.78)  0.28 (-0.48, 1.04) | 0.215  0.470 |
|  |  |  |  |
| Interaction effects of child’s sex and child’s age on the association between intersection density and MVPA | Sex  Age | 15.21 (-71.61, 102.0)  4.89 (-20.80, 30.58) | 0.732  0.709 |
|  |  |  |  |
| Interaction effects of child’s sex and child’s age on the association between number of parks in 1 km buffer and MVPA | Sex  Age | -0.77 (-3.72, 2.18)  -0.04 (-0.95, 0.86) | 0.608  0.926 |
| Interaction effects of child’s sex and child’s age on the association between PA equipment at home and MVPA | Sex  Age | -0.13 (-4.03, 3.77)  0.22 (-0.99, 1.43) | 0.948  0.718 |
| Interaction effects of child’s sex and child’s age on the association between perceived traffic and MVPA | Sex  Age | 1.65 (-9.90, 13.20)  0.83 (-2.73, 4.39) | 0.780  0.648 |
| Interaction effects of child’s sex and child’s age on the association between perceived crime and MVPA | Sex  Age | 1.67 (-6.74, 10.08)  0.94 (-1.78, 3.65) | 0.698  0.499 |

**a -**  generalised additive mixed model **(GAMM) with gaussian distribution used for child’s MVPA, adjusted for same covariates as used for Direct effects. All models also adjusted for census block group cluster id and accelerometer wear-time;** b = regression coefficient; CI = confidence interval. Play space score was measured using Informal Play Space audit tool; Positive AT score – a MAPS subscale measuring positive characteristics of the neighbourhood for promoting active transport (AT); Park PA facilities score - measured using the Environmental Assessment of Public Recreational Spaces (EAPRS) audit tool; Land use mix – measured using ‘MAPS Destination Land Use (DLU) positive overall’ score; Residential density – measured using ‘MAPS Res_Density_Mix_recode’ score.

None of the two-way interaction effects of child’s age and sex on the association between environmental attributes and MVPA were statistically significant.


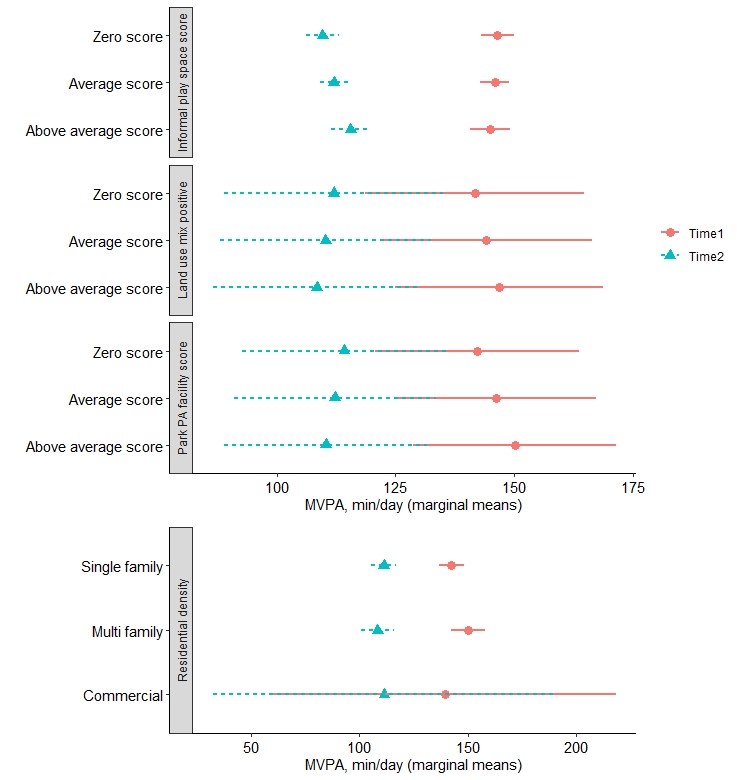


**Figure S4-1. Marginal means for moderate-to-vigorous physical activity (MVPA) at Times 1 and 2 where there were significant interaction effects of environmental attributes on the association between time and child’s MVPA**
